# Associations Among the Big Five Personality Traits, Maladaptive Cognitions, and Internet Addiction Across Three Time Measurements in 3 Months During the COVID-19 Pandemic

**DOI:** 10.3389/fpsyg.2021.654825

**Published:** 2021-05-20

**Authors:** Yu Tian, Yanli Zhao, Fengling Lv, Ningbo Qin, Peipei Chen

**Affiliations:** ^1^Department of Marxism, Qingdao University of Science and Technology, Qingdao, China; ^2^Department of Psychology, Shandong Normal University, Jinan, China

**Keywords:** Internet addiction, maladaptive cognitions, Big Five personality traits, cross-lagged panel design, cognitive–behavioral model

## Abstract

The present study examined the longitudinal association among the Big Five personality traits, maladaptive cognitions, and Internet addiction during the COVID-19 pandemic. A total of 481 Chinese university students (247 men; mean age = 20.31 years) were surveyed three times (interval of 1 month) by using the Chinese version of the Big Five Personality Traits Scale, Maladaptive Cognitions Scale, and Internet Addiction Scale. The results of a cross-lagged panel analysis highlighted that (i) extraversion, agreeableness, conscientiousness, and openness were negatively associated with maladaptive cognitions and Internet addiction, whereas neuroticism was found to be positively associated with maladaptive cognitions and Internet addiction across time; (ii) associations among the Big Five personality traits, maladaptive cognitions, and Internet addiction were dynamic and bidirectional; and (iii) maladaptive cognitions played mediating roles in extraversion, agreeableness, conscientiousness, openness, and Internet addiction across time. The Big Five personality traits, maladaptive cognitions, and Internet addiction predicted each other across time, and maladaptive cognitions were likely to be the key mediating factor in the associations between the Big Five personality traits and Internet addiction, which supported and expanded the Davis’ cognitive–behavioral model.

## Introduction

According to the 45th China Statistical Report on Internet Development, more than 904 million Chinese people spent an average of 30.8 h every week on the Internet to shop, play games, communicate with others, and perform other daily life activities (China Internet Network Information Center [CNNIC], 2020). However, spending too much time on the Internet leads to individuals getting affected by Internet addiction [also referred to as pathological Internet use (PIU) in other studies], which has been defined as excessive or compulsive Internet use and preoccupation with and loss of control over this use (Davis, 2001). Numerous recent studies have reported that people, especially university students, have been using the Internet more during the COVID-19 pandemic to mitigate their anxiety levels, resulting in an increased level of Internet addiction (Garcia-Priego et al., 2020; Jiang, 2020; Nagaur, 2020). Previous studies have reported Internet addiction to be positively associated with numerous negative outcomes, including decreased academic achievement (Stavropoulos et al., 2013), emotional disorders (Kim et al., 2017), sleep disorders (Ladner et al., 2010), and even suicidal ideation and behavior in some serious cases (Steinbüchel et al., 2017; Sami et al., 2018). Therefore, a study of factors affecting Internet addiction during the COVID-19 pandemic is warranted to provide implications for the prevention of Internet addiction during this unprecedented time.

Previous studies have suggested that the Big Five personality traits, namely, extraversion, agreeableness, conscientiousness, neuroticism, and openness, are significantly associated with Internet addiction (Zhou et al., 2017; Hou et al., 2018; Kircaburun and Griffiths, 2018; Agbaria and Bdier, 2020; Arpaci and Unver, 2020). Recently, a meta-analysis review study proposed that extraversion, agreeableness, conscientiousness, and openness are negatively associated with Internet addiction, whereas neuroticism is positively associated with Internet addiction (Kayiş et al., 2016). Although previous research has highlighted clear associations between the Big Five personality traits and Internet addiction, several gaps still exist in the relevant literature. First, a majority of studies examining the effects of the Big Five personality traits on Internet addiction had a cross-sectional design, which made it difficult to draw causal inferences. Therefore, more studies with a longitudinal design are warranted to further validate the results of previous studies. Second, the mechanism through which the Big Five personality traits affect Internet addiction has received limited research attention. Although some studies have highlighted coping style (Zhou et al., 2017), self-liking (Kircaburun and Griffiths, 2018), and family function (Hou et al., 2018) to mediate associations between the Big Five personality traits and Internet addiction, more mediating factors should be investigated. Finally, no study has investigated the effects of Internet addiction on the Big Five personality traits; the literature has only suggested that Internet addiction exerts effects on individuals’ personality traits, such as shyness and depression (Tian et al., 2017; Soulioti et al., 2018). Therefore, the effects of Internet addiction on individuals’ Big Five personality traits merit further research. Considering the aforementioned limitations, the present study provided an in-depth exploration of this topic.

## Bidirectional Associations Between the Big Five Personality Traits and Internet Addiction

Previous studies have reported that extraversion, agreeableness, conscientiousness, and openness are negatively associated with the positive predictors of Internet addictions, such as low self-esteem (Wang et al., 2012), loneliness (Buecker et al., 2020), depression (Bunevicius et al., 2008), and social anxiety (Lee et al., 2014), whereas neuroticism was found to be positively associated with these predictors. Therefore, most studies have suggested that extraversion, agreeableness, conscientiousness, and openness are significantly negative predictors of Internet addiction, whereas neuroticism is a significantly positive predictor of Internet addiction (Kayiş et al., 2016). However, some studies have proposed different conclusions; for example, positive or no associations were found between openness and Internet addiction (Rahmani and Lavasani, 2011; Servidio, 2014) and between extraversion and Internet addiction (Rahmani and Lavasani, 2011; Andreassen et al., 2013). Therefore, further investigation, especially longitudinal studies, on the effects of the Big Five personality traits on Internet addiction is required.

Internet addiction tends to be a predictor of the Big Five personality traits. Although individuals’ personality traits are stable, they may fluctuate within a certain range (Asendorpf and Aken, 2010). Numerous studies have demonstrated that Internet addiction may affect individuals’ personality traits, such as loneliness (Yao and Zhong, 2014; Tian et al., 2017), depression (Bengü and Ahmet, 2018), and self-esteem issues (Fioravanti et al., 2012). Moreover, the Big Five personality traits have been significantly associated with individuals’ loneliness, depression, and self-esteem (Bunevicius et al., 2008; Wang et al., 2012; Buecker et al., 2020), thereby, indicating that the Big Five personality traits can be the outcome variables of Internet addiction. However, an exhaustive review of the relevant literature revealed that the effects of Internet addiction on individuals’ Big Five personality traits are yet to be studied; therefore, the present study examined these effects.

## The Bidirectional Mediating Roles of Maladaptive Cognitions

The cognitive–behavioral model was employed in our study as the theoretical model to clarify associations and the mechanism through which the Big Five personality traits affect Internet addiction. The model indicated that psychopathology (such as substance dependence, depression, and social anxiety) is a distal necessary cause of symptoms of Internet addiction (Davis, 2001). However, psychopathology alone cannot lead to individuals getting affected by Internet addiction, and it is just a necessary element in the etiology of Internet addiction. Maladaptive cognitions, defined as cognitive distortions about the self (such as “I am good only on the Internet”) and the world (such as “The Internet is the only place where I am respected”), are the proximal sufficient causes of Internet addiction. The distal necessary causes may affect Internet addiction through the proximal sufficient causes. Previous studies have highlighted that the Big Five personality traits are significantly associated with depression (Langguth et al., 2007) and social anxiety (Kaplan et al., 2015); therefore, they constitute the distal necessary causes, in turn, suggesting that the Big Five personality traits may also affect Internet addiction through maladaptive cognitions. Although numerous studies have found that individuals’ personality traits, such as shyness (Tian et al., 2015), motivation, stress, loneliness, and depression (Lu and Yeo, 2015), can affect Internet addiction through maladaptive cognition, no study has examined the mediating roles of maladaptive cognitions in the associations between the Big Five personality traits and Internet addiction; therefore, this study intends to fill the gaps in research.

The cognitive–behavioral model suggests that the symptoms of Internet addiction could get exhibited through vicious cycles, which can further affect individuals’ personality (Davis, 2001). For example, many studies have indicated that Internet addiction can exert a negative effect on individuals’ shyness (Tian et al., 2017), self-esteem (Mo et al., 2020), and depression (Soulioti et al., 2018), thereby highlighting that Internet addiction can also exert negative effects on individuals’ Big Five personality traits. In addition, the cognitive–behavioral model explained the mechanism through which Internet addiction affects individuals’ Big Five personality traits. The model emphasized that the core symptoms of Internet addiction constitute maladaptive cognition, and individuals with Internet addiction are disposed to regard the Internet as the only place where they feel good about themselves (Davis, 2001). More specifically, individuals with maladaptive cognitions tend to spend more time or money on the Internet and less time on activities that they may have found pleasurable before. When an individual reduces face-to-face interactions with their friends or family members, they become socially isolated and experience weak social support and increasing interpersonal issues, which may affect their personality traits (Tian et al., 2017; Soulioti et al., 2018). On the basis of vicious cycles through which Internet addiction operates, maladaptive cognitions also tend to be a key factor determining the effect of Internet addiction on individuals’ Big Five personality traits. Therefore, this point of view was also verified in the present study.

## The COVID-19 Pandemic

Studies have highlighted that the level of individuals’ Internet addiction has increased during the COVID-19 pandemic (Garcia-Priego et al., 2020; Jiang, 2020; Nagaur, 2020). This is because the absence of definite answers to the questions of when the pandemic is going to end and how one can cope with this challenging time may lead individuals to experience more anxiety, depression, and other negative emotions (Özdin and Özdin, 2020). To attenuate the effect of such negative emotions, individuals tend to use the Internet more to release these emotions; however, spending too much time on the Internet may lead to an increased level of Internet addiction (Garcia-Priego et al., 2020; Jiang, 2020). Moreover, prohibitions/recommendations, such as staying at home as much as possible, make it difficult for individuals to have face-to-face communication with each other; therefore, individuals spend more time on the Internet to maintain social ties, which could, in turn, intensify their Internet addiction (Nagaur, 2020). Because of the specific characteristics of this period, the associations between, and the mechanism through which, the Big Five personality traits affect Internet addiction are different. For example, extraversion, agreeableness, conscientiousness, and openness have been found to be negatively associated with anxiety, depression, and other negative emotions (Bunevicius et al., 2008; Wang et al., 2012; Lee et al., 2014), whereas neuroticism is positively associated with these negative emotions. These associations may lead to stronger negative or positive connections between the Big Five personality traits and Internet addiction. Because maladaptive cognitions are the proximal sufficient causes of Internet addiction, stronger associations may be established because of the increased level of maladaptive cognitions. However, these hypotheses required validation in the current study.

## The Present Study

The present study examined reciprocal associations among the Big Five personality traits, maladaptive cognitions, and Internet addiction by using a longitudinal design and tested the bidirectional mediating roles of maladaptive cognitions in the association between the Big Five personality traits and Internet addiction. Drawing from the aforementioned literature review, we proposed the following hypotheses: (i) Extraversion, maladaptive cognitions, and Internet addiction can predict each other across time, and maladaptive cognitions play bidirectional mediating roles in the association between extraversion and Internet addiction. (ii) Agreeableness, maladaptive cognitions, and Internet addiction can predict each other across time, and maladaptive cognitions play bidirectional mediating roles in the association between agreeableness and Internet addiction. (iii) Conscientiousness, maladaptive cognitions, and Internet addiction can predict each other across time, and maladaptive cognitions play bidirectional mediating roles in the association between conscientiousness and Internet addiction. (iv) Neuroticism, maladaptive cognitions, and Internet addiction can predict each other across time, and maladaptive cognitions play bidirectional mediating roles in the association between neuroticism and Internet addiction. (v) Openness, maladaptive cognitions, and Internet addiction can predict each other across time, and maladaptive cognitions play bidirectional mediating roles in the association between openness and Internet addiction. The aforementioned hypotheses were tested using a cross-lagged panel design in which three measurement points of each studied variable were employed.

## Materials and Methods

### Participants and Procedure

All students recruited in the present study belonged to the same university, which was located in the northeast of China. Three measurements were conducted during the COVID-19 pandemic at 1-month intervals. During the first measurement, 734 valid questionnaires were collected, of which 390 were completed by male students. During the second measurement, 656 valid questionnaires were collected, of which 345 were completed by male students. During the third measurement, 599 valid questionnaires were collected, of which 304 were completed by male students. A total of 481 students finished all the three measurements, and their responses were utilized for subsequent data analysis. More specifically, the final sample comprised 247 male students and 234 female students, and the mean age of the final sample was 20.31 [standard deviation (SD) = 1.63] years. Some students could not take the surveys because of illness, because they did not receive the message appropriately, or because of the inconvenient network links, which made them give up the survey. A series of independent sample *t*-tests were performed to estimate differences between participating students and non-participating students in the studied variables. No differences were found.

All the three measurements were conducted online because of prohibitions/recommendations to stay at home, and the survey was conducted through three steps. First, teachers sent a message to the students, requesting them to participate in the study’s online survey. The students were informed that the survey comprised three measurements that were separated by 1 month each. All the students provided their consent to take part in the survey, and only those who finished all the three measurements were compensated with gifts. Moreover, students were informed that the scores of the survey would not be recorded as their final performance for that term and would be kept confidential. Second, the teachers organized for the students to complete each of the measurements after an online class, and all the students were required to respond to the questionnaires in the time provided. All the teachers were required to be present online during the measurement and answer any questions that came from the students. Third, the teachers briefed the students regarding the primary objective of this survey during the final measurement and distributed gifts among them as rewards.

### Measures

#### Big Five Personality Traits

The Chinese version of the Big Five Inventory was employed to measure the participants’ Big Five personality traits (John et al., 1991; Carciofo et al., 2016). The revised questionnaire comprised 44 items, with an eight-item extraversion subscale (e.g., “I am talkative”), a nine-item agreeableness subscale (e.g., “I am helpful and unselfish”), a nine-item conscientiousness subscale (e.g., “I am rigorous and earnest at work”), an eight-item neuroticism subscale (e.g., “I am depressive and melancholy”), and a 10-item openness subscale (e.g., “I am original and constantly coming up with new ideas”). The revised scale employed a five-point Likert from 1 (strongly disagree) to 5 (strongly agree) to estimate the level of each personality trait among the respondents. Cronbach’s α values for the extraversion subscale at the three time points in the present study were 0.69, 0.64, and 0.68, respectively. Moreover, the values, respectively, were 0.74, 0.77, and 0.73 for the agreeableness subscale; 0.77, 0.75, and 0.77 for the conscientiousness subscale; 0.70, 0.69, and 0.71 for the neuroticism subscale; 0.73, 0.74, and 0.77 for the openness sub-scale; and 0.77, 0.79, and 0.81 for the total scale.

#### Maladaptive Cognitions

The short Chinese version of the Online Cognition Scale (OCS) was utilized to measure individuals’ maladaptive cognitions (Davis et al., 2002; Liang, 2008). This scale was related to the degree of comfort they experienced on the Internet (e.g., “I feel very safe when I on the Internet”), their desire and control capability to use the Internet (e.g., “I often use the Internet beyond the limited degree”), and their use of the Internet as an escape from stress (e.g., “I often surf the Internet to avoid doing unpleasant things”). This scale that was used to estimate the level of individuals’ maladaptive cognitions comprised 14 items that were responded to on a four-point Likert scale ranging from 1 (strongly disagree) to 4 (strongly agree). Cronbach’s α values for the scale at the three time points in the present study were 0.92, 0.95, and 0.95, respectively.

#### Internet Addiction

The Chinese version of the Internet Addiction Test (IAT) was employed to measure individuals’ Internet addiction (Young, 1998; Bai and Fan, 2005), which was related to compulsive Internet access and withdrawal reaction (e.g., “*Irrespective of how tired I am, I always feel energetic when I surf the Internet*”), Internet addiction tolerance (e.g., “*I find myself spending an increasing amount of time on the Internet*”), time management questions (e.g., “*Because of the Internet, my leisure time has been reduced*”), and interpersonal and health problems (e.g., “*I’ve been told more than once that I spend too much time online*”). The scale comprised 19 items that were responded to on a four-point Likert scale ranging from 1 (strongly disagree) to 4 (strongly agree) that was used to estimate the level of individuals’ Internet addiction. Cronbach’s α values for the scale at the three time points in the present study were 0.94, 0.96, and 0.97, respectively.

### Statistical Analysis

SPSS 20.0 was employed to conduct a correlation analysis (Pearson’s correlation) and difference tests (repeated measurement analysis of variance). Additionally, Mplus 7.0 was used to conduct structural equation modeling (SEM) of path analysis (cross-lagged model). More specifically, the total scores of extraversion, agreeableness, conscientiousness, neuroticism, openness, maladaptive cognitions, and Internet addiction were used as observed variables in all the analyses. Within cross-lagged models, the autoregressive effect between time-adjacent measurements was employed to evaluate the stability of the studied variables across time (e.g., T1 extraversion predicts T2 extraversion, and T2 extraversion predicts T3 extraversion); unidirectional effects between the two variables were used as causal associations between the studied variables (e.g., T1 extraversion predicts T2 Internet addiction, and T2 extraversion predicts T3 Internet addiction); and bidirectional effects between the two variables were used as cross-lagged associations between the studied variables (e.g., T1 extraversion predicts T2 Internet addiction, and T1 Internet addiction predicts T2 extraversion). Bootstrapping was conducted with 1,000 bootstrap samples to determine the mediating role of maladaptive cognitions in the association between the Big Five personality traits and Internet addiction. Furthermore, Hu and Bentler’s (1999) goodness of fit indices of chi-squared (χ^2^) statistic, degree of freedom (df), Tucker–Lewis index (TLI), comparative fit index (CFI), and root-mean-square error of approximation (RMSEA) were used to assess each of the SEMs.

## Results

### Correlation Analysis and Difference Tests

Table 1 presents the association between all the studied variables across time. The association among T1, T2, and T3 extraversion ranged from 0.64 to 0.68, and that among T1, T2, and T3 agreeableness ranged from 0.55 to 0.65. The association among T1, T2, and T3 conscientiousness ranged from 0.58 to 0.72, and that among T1, T2, and T3 neuroticism ranged from 0.57 to 0.65. The association among T1, T2, and T3 openness ranged from 0.53 to 0.54, and that among T1, T2, and T3 maladaptive cognitions ranged from 0.50 to 0.59. The association among T1, T2, and T3 Internet addiction ranged from 0.53 to 0.63. The associations among the measures of extraversion, agreeableness, conscientiousness, neuroticism, openness, maladaptive cognitions, and Internet addiction were in the expected direction. That is, extraversion, agreeableness, conscientiousness, and openness were negatively associated with maladaptive cognitions and Internet addiction, whereas neuroticism was positively associated with maladaptive cognitions and Internet addiction. Furthermore, maladaptive cognitions and Internet addiction were positively associated with each other.

**TABLE 1:** The associations among the Big Five personality traits, maladaptive cognitions, and Internet addiction across time.

Variables	1	2	3	4	5	6	7	8	9	10	11	12	13	14	15	16	17	18	19	20	21
1. T1 extraversion	1																				
2. T1 agreeableness	0.36**	1																			
3. T1 conscientiousness	0.50**	0.52**	1																		
4. T1 neuroticism	–0.47	−0.51**	−0.44**	1																	
5. T1 openness	0.53**	0.49**	0.52**	–0.38	1																
6. T1 MC	−0.21**	−0.39**	−0.36**	0.32**	−0.20**	1															
7. T1 Internet addiction	−0.29**	−0.38**	−0.46**	0.36**	−0.28**	0.67**	1														
8. T2 extraversion	0.68**	0.35**	0.39**	−0.43**	0.39**	−0.25**	−0.30**	1													
9. T2 agreeableness	0.28**	0.61**	0.37**	−0.38**	0.29**	−0.37**	−0.41**	0.51**	1												
10. T2 conscientiousness	0.38**	0.42**	0.65**	−0.39**	0.37**	−0.33**	−0.42**	0.59**	0.58**	1											
11. T2 neuroticism	−0.37**	−0.36**	−0.34**	0.59**	−0.27**	0.30**	0.33**	−0.57**	−0.55**	−0.59**	1										
12. T2 openness	0.38**	0.38**	0.35**	−0.30**	0.53**	−0.20**	−0.26**	0.58**	0.55**	0.55**	−0.34**	1									
13. T2 MC	−0.25**	−0.30**	−0.35**	0.29**	−0.20**	0.59**	0.53**	−0.31**	−0.38**	−0.38**	0.41**	−0.21**	1								
14. T2 Internet addiction	−0.29**	−0.36**	−0.42**	0.36**	−0.25**	0.54**	0.63**	−0.37**	−0.43**	−0.47**	0.44**	−0.30**	0.70**	1							
15. T3 extraversion	0.64**	0.29**	0.38**	−0.36**	0.39**	−0.23**	−0.28**	0.66**	0.36**	0.46**	−0.47**	0.43**	−0.26**	−0.28**	1						
16. T3 agreeableness	0.28**	0.55**	0.38**	−0.33**	0.32**	−0.37**	−0.43**	0.40**	0.65**	0.49**	−0.46**	0.42**	−0.40**	−0.43**	0.55**	1					
17. T3 conscientiousness	0.37**	0.41**	0.58**	−0.33**	0.37**	−0.33**	−0.42**	0.50**	0.48**	0.72**	−0.51**	0.42**	−0.43**	−0.45**	0.63**	0.66**	1				
18. T3 neuroticism	−0.35**	−0.34**	−0.34**	0.57**	−0.25**	0.29**	0.36**	−0.49**	−0.44**	−0.49**	0.65**	−0.36**	0.36**	0.40**	−0.60**	−0.57**	−0.61**	1			
19. T3 openness	0.41**	0.38**	0.41**	−0.28**	0.54**	−0.24**	−0.31**	0.44**	0.41**	0.45**	−0.40**	0.53**	−0.30**	−0.29**	0.63**	0.64**	0.62**	−0.43**	1		
20. T3 MC	−0.19**	−0.25**	−0.28**	0.23**	−0.20**	0.50**	0.49**	−0.28**	−0.39**	−0.32**	0.33**	−0.23**	0.57**	0.53**	−0.30**	−0.47**	−0.45**	0.41**	−0.28**	1	
21. T3 Internet addiction	−0.25**	−0.35**	−0.36**	0.30**	−0.26**	0.46**	0.54**	−0.35**	−0.48**	−0.41**	0.38**	−0.36**	0.54**	0.64**	−0.31**	−0.52**	−0.50**	0.47**	−0.34**	0.73**	1

**p < 0.01. MC, maladaptive cognitions.

The results of the repeated measurement analysis of variance indicated a significant difference in Internet addiction across time, and no other differences were revealed. Furthermore, the results of pairwise comparisons indicated no significant difference in extraversion, conscientiousness, and maladaptive cognitions across time; however, agreeableness and openness decreased at T2, neuroticism increased at T2, and Internet addiction increased at T3 (see Table 2).

**TABLE 2:** Pairwise comparisons of the Big Five personality traits, maladaptive cognitions, and Internet addiction across time.

Variables	T1 (*M* ± *SD*)	T2 (*M* ± *SD*)	T3 (*M* ± *SD*)	*F* (1, 480)	*P*
Extraversion	26.01 ± 4.41	26.05 ± 4.25	26.20 ± 4.58	1.09	0.30
Agreeableness	34.45 ± 4.86	33.72 ± 5.14	34.17 ± 4.64	1.75	0.19
Conscientiousness	29.91 ± 5.03	29.76 ± 5.07	30.08 ± 5.33	0.57	0.45
Neuroticism	21.55 ± 4.80	21.99 ± 4.60	21.89 ± 4.84	2.75	0.10
Openness	34.76 ± 4.98	34.19 ± 5.20	34.33 ± 5.47	3.57	0.06
Maladaptive cognitions	33.70 ± 10.15	34.00 ± 11.12	34.69 ± 11.89	3.81	0.05
Internet addiction	37.70 ± 10.80	38.00 ± 11.87	38.90 ± 13.34	4.95	0.03

### Cross-Lagged Associations Among the Big Five Personality Traits, Maladaptive Cognitions and Internet Addiction

**FIGURE 1:**
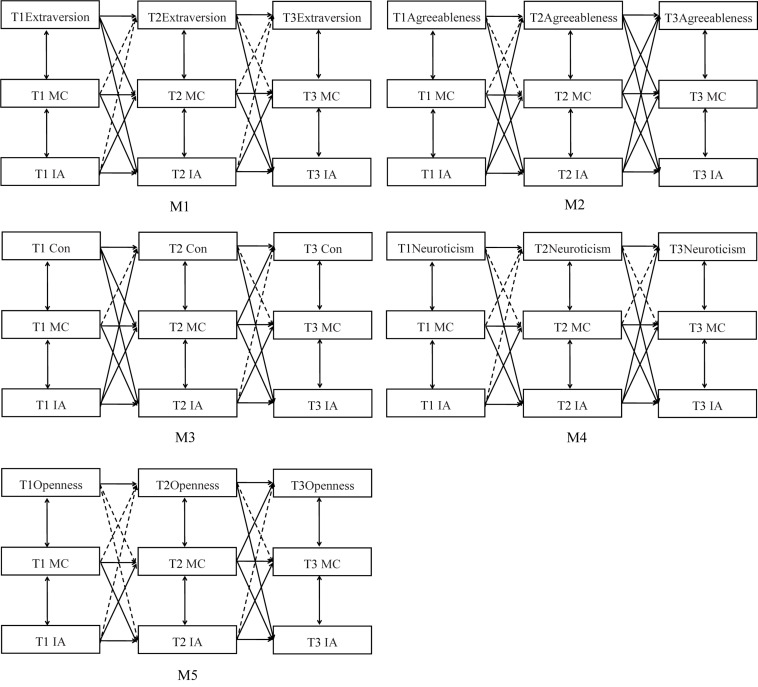
Cross-lagged associations among the Big Five personality traits, maladaptive cognitions, and Internet addiction. Thick lines represent significant paths, and dashed lines represent non-significant paths.

To verify the aforementioned hypothesis, five models (M1–M5) were built to test cross-lagged associations among the Big Five personality traits, maladaptive cognitions, and Internet addiction. First, M1 was built to examine cross-lagged associations among extraversion, maladaptive cognitions, and Internet addiction across time. The results of path analysis highlighted that M1 achieved good model fit indexes (see M1 in Table 3). Moreover, the results indicated that T1 extraversion could negatively predict T2 maladaptive cognitions, extraversion could negatively predict Internet addiction across time, maladaptive cognitions and Internet addiction could positively predict each other across time, and all the autoregressive effects of extraversion, maladaptive cognitions, and Internet addiction were significant (see M1 in Table 3 and Figure 1). The bootstrapping results suggested that T1 extraversion could affect T3 Internet addiction through the mediating roles of T2 maladaptive cognitions (95%: −0.031 to −0.002) (see Table 4).

**TABLE 3:** Fit statistics for the model comparison of cross-lagged associations among the Big Five personality traits, maladaptive cognitions, and Internet addiction.

Models	χ^2^	*df*	*P*	TLI	CFI	RMSEA
M1	98.32	9	<0.001	0.84	0.96	0.14
M2	65.81	9	<0.001	0.90	0.97	0.12
M3	52.07	9	<0.001	0.92	0.98	0.10
M4	83.57	9	<0.001	0.86	0.96	0.13
M5	96.33	9	<0.001	0.82	0.95	0.14

*The larger number of the published studies with this method had suggested that the model fit indices of the present study was acceptable (e.g., Hu and Bentler, 1999).*

**TABLE 4:** Regression coefficients of cross-lagged associations among extraversion, maladaptive cognitions, and Internet addiction within M1.

Items	T1 → T2			T2 → T3		
Paths	β	*SE*	*P*	β	*SE*	*P*
Autoregressive effect of extraversion	0.65	0.03	<0.001	0.64	0.04	<0.001
Autoregressive effect of maladaptive cognitions	0.42	0.05	<0.001	0.38	0.06	<0.001
Autoregressive effect of Internet addiction	0.45	0.05	<0.001	0.47	0.06	<0.001
Extraversion → maladaptive cognitions	−0.10	0.09	0.008	−0.07	0.11	>0.05
Extraversion → Internet addiction	−0.12	0.10	<0.001	−0.12	0.12	<0.001
Maladaptive cognitions → Internet addiction	0.21	0.06	<0.001	0.17	0.06	<0.001
Internet addiction → maladaptive cognitions	0.23	0.05	<0.001	0.24	0.05	<0.001
Internet addiction → extraversion	−0.06	0.02	>0.05	0.01	0.02	>0.05
Maladaptive cognitions → extraversion	−0.07	0.02	>0.05	−0.07	0.02	>0.05

Second, M2 was developed to study cross-lagged associations among agreeableness, maladaptive cognitions, and Internet addiction across time. The results of path analysis indicated that M2 achieved good model fit indexes (see M2 in Table 3). Moreover, the results highlighted that T2 agreeableness could negatively predict T3 maladaptive cognitions, and agreeableness and Internet addiction could negatively predict each other across time. Additionally, maladaptive cognitions and Internet addiction could positively predict each other across time, and T2 maladaptive cognitions could negatively predict T3 agreeableness. All the autoregressive effects of agreeableness, maladaptive cognitions, and Internet addiction were significant (see M2 in Table 3 and Figure 1). The bootstrapping results have suggested that T1 Internet addiction could affect T3 agreeableness through the mediating roles of T2 maladaptive cognitions (95%: −0.051 to −0.002) (see Table 5).

**TABLE 5:** Regression coefficients of cross-lagged associations among agreeableness, maladaptive cognitions, and Internet addiction within M2.

Items	T1 → T2			T2 → T3		
Paths	β	*SE*	*P*	β	*SE*	*P*
Autoregressive effect of agreeableness	0.53	0.05	<0.001	0.57	0.04	<0.001
Autoregressive effect of maladaptive cognitions	0.41	0.06	<0.001	0.36	0.07	<0.001
Autoregressive effect of Internet addiction	0.46	0.07	<0.001	0.43	0.07	<0.001
Agreeableness → maladaptive cognitions	−0.05	0.12	>0.05	−0.16	0.11	0.001
Agreeableness → Internet addiction	−0.11	0.12	0.020	−0.24	0.12	<0.001
Maladaptive cognitions → Internet addiction	0.19	0.06	<0.001	0.15	0.07	0.017
Internet addiction → maladaptive cognitions	0.24	0.07	<0.001	0.21	0.06	0.001
Internet addiction → agreeableness	−0.18	0.02	<0.001	−0.11	0.02	0.037
Maladaptive cognitions → agreeableness	−0.04	0.03	>0.05	−0.11	0.02	0.028

Third, M3 was built to assess cross-lagged associations among conscientiousness, maladaptive cognitions, and Internet addiction across time. The results of path analysis indicated that M3 achieved good model fit indexes (see M3 in Table 3). Moreover, the results indicated that T1 conscientiousness could negatively predict T2 maladaptive cognitions, and conscientiousness could negatively predict Internet addiction across time. Maladaptive cognitions and Internet addiction could positively predict each other across time, and T2 maladaptive cognitions could negatively predict T3 conscientiousness. Furthermore, T1 Internet addiction could negatively predict T2 conscientiousness, and all the autoregressive effects of conscientiousness, maladaptive cognitions, and Internet addiction were significant (see M3 in Table 3 and Figure 1). The bootstrapping results revealed that T1 conscientiousness could affect T3 Internet addiction through the mediating roles of T2 maladaptive cognitions (95%: -0.035 to -0.002), and T1 Internet addiction could affect T3 conscientiousness through the mediating roles of T2 maladaptive cognitions (95%: −0.055 to −0.01) (see Table 6).

**TABLE 6:** Regression coefficients of cross-lagged associations among conscientiousness, maladaptive cognitions, and Internet addiction within M3.

Items	T1 → T2			T2 → T3		
Paths	β	*SE*	*P*	*B*	*SE*	*P*
Autoregressive effect of conscientiousness	0.57	0.05	<0.001	0.64	0.04	<0.001
Autoregressive effect of maladaptive cognitions	0.41	0.06	<0.001	0.38	0.07	<0.001
Autoregressive effect of Internet addiction	0.42	0.07	<0.001	0.46	0.07	<0.001
Conscientiousness → maladaptive cognitions	−0.11	0.09	0.006	−0.06	0.11	>0.05
Conscientiousness → Internet addiction	−0.16	0.08	<0.001	−0.13	0.11	0.002
Maladaptive cognitions → Internet addiction	0.20	0.06	<0.001	0.17	0.08	0.008
Internet addiction → maladaptive cognitions	0.21	0.07	0.001	0.23	0.06	<0.001
Internet addiction → conscientiousness	−0.14	0.02	0.003	−0.04	0.02	>0.05
Maladaptive cognitions → conscientiousness	−0.03	0.02	>0.05	−0.16	0.02	0.001

Fourth, M4 was developed to evaluate the cross-lagged associations among neuroticism, maladaptive cognitions, and Internet addiction across time. The results of path analysis revealed that M4 achieved good model fit indexes (see M4 in Table 3). Furthermore, the results demonstrated that neuroticism could positively predict Internet addiction across time, maladaptive cognitions and Internet addiction could positively predict each other across time, T2 Internet addiction could positively predict T3 neuroticism, and all the autoregressive effects of neuroticism, maladaptive cognitions, and Internet addiction were significant (see M4 in Table 3 and Figure 1) (see Table 7).

**TABLE 7:** Regression coefficients of cross-lagged associations among neuroticism, maladaptive cognitions, and Internet addiction within M4.

Items	T1 → T2			T2 → T3		
Paths	β	*SE*	*P*	*B*	*SE*	*P*
Autoregressive effect of neuroticism	0.54	0.05	<0.001	0.58	0.05	<0.001
Autoregressive effect of maladaptive cognitions	0.41	0.06	<0.001	0.37	0.07	<0.001
Autoregressive effect of Internet addiction	0.45	0.08	<0.001	0.48	0.07	<0.001
Neuroticism → maladaptive cognitions	0.08	0.11	>0.05	0.07	0.13	>0.05
Neuroticism → Internet addiction	0.13	0.11	0.002	0.11	0.13	0.014
Maladaptive cognitions → Internet addiction	0.20	0.06	<0.001	0.16	0.08	0.012
Internet addiction → maladaptive cognitions	0.23	0.07	<0.001	0.24	0.06	<0.001
Internet addiction → neuroticism	0.10	0.02	>0.05	0.11	0.02	0.019
Maladaptive cognitions → neuroticism	0.07	0.02	>0.05	0.04	0.02	>0.05

Fifth, M5 was built to examine cross-lagged associations among openness, maladaptive cognitions, and Internet addiction across time. The results of path analysis highlighted that M5 achieved good model fit indexes (see M5 in Table 3). Furthermore, the results revealed that T2 openness could negatively predict T3 Internet addiction, T2 maladaptive cognitions could negatively predict T3 openness, maladaptive cognitions and Internet addiction could positively predict each other across time, and all the autoregressive effects of openness, maladaptive cognitions, and Internet addiction were significant (see M5 in Table 3 and Figure 1). The bootstrapping results have suggested that T1 Internet addiction could affect T3 openness through the mediating roles of T2 maladaptive cognitions (95%: −0.08 to −0.015) (see Table 8).

**TABLE 8:** Regression coefficients of cross-lagged associations among openness, maladaptive cognitions, and Internet addiction within M5.

Items	T1 → T2			T2 → T3		
Paths	β	*SE*	*P*	β	*SE*	*P*
Autoregressive effect of openness	0.50	0.04	<0.001	0.49	0.05	<0.001
Autoregressive effect of maladaptive cognitions	0.42	0.06	<0.001	0.39	0.07	<0.001
Autoregressive effect of Internet addiction	0.46	0.07	<0.001	0.45	0.07	<0.001
Openness → maladaptive cognitions	−0.04	0.09	>0.05	−0.09	0.11	>0.05
Openness → Internet addiction	−0.07	0.10	>0.05	−0.18	0.11	<0.001
Maladaptive cognitions → Internet addiction	0.21	0.06	<0.001	0.18	0.07	0.003
Internet addiction → maladaptive cognitions	0.24	0.07	<0.001	0.23	0.07	<0.001
Internet addiction → openness	−0.10	0.03	>0.05	−0.00	0.03	>0.05
Maladaptive cognitions → openness	−0.04	0.03	>0.05	−0.20	0.03	0.001

## Discussion

The present study is the first to test bidirectional associations among the Big Five personality traits, maladaptive cognitions, and Internet addiction during the COVID-19 pandemic period by using a longitudinal design. The results suggested that the associations among the Big Five personality traits, maladaptive cognitions, and Internet addiction were dynamic and bidirectional. Moreover, the results indicated that maladaptive cognitions played important mediating roles in the associations among extraversion, agreeableness, conscientiousness, openness, and Internet addiction across time. These results not only verified the conclusions of previous studies through a cross-section design but also tested the mediating roles of maladaptive cognitions in the associations between the Big Five personality traits and Internet addiction. These associations and their influential mechanism were further clarified as follows.

First, the present study demonstrated that extraversion affected individuals’ Internet addiction unidirectionally. More specially, extraversion affected not only individuals’ Internet addiction directly but also their Internet addiction through maladaptive cognitions indirectly, which supported the study’s cognitive–behavioral model. Extraversion has been defined as the intensity and volume of people’s social interaction, and it indicates whether people can act with competition and self-confidence (McCrae and Costa, 1997). According to the aforementioned definition of extraversion, individuals with increased social interaction and higher level of competition or self-confidence exhibit a lower level of Internet addiction, which is consistent with previous findings (Baturay and Toker, 2019). Additionally, previous studies have reported that individuals with social problems, which was negatively related with extraversion, demonstrated more maladaptive cognitions with regard to Internet use (Davis, 2001; Tian et al., 2015; Gao et al., 2019). Moreover, the present study found extraversion to negatively predict Internet addiction through maladaptive cognitions, which further verified and expanded the results of previous studies. However, some previous studies have identified no or a positive association between extraversion and Internet addiction (Rahmani and Lavasani, 2011; Andreassen et al., 2013). These inconsistent results can be attributed to the employment of different samples or instruments; for example, one study found that the Chinese population tends to develop a higher level of Internet addiction than people in Western countries (Hu et al., 2012). Therefore, the present study’s results should be verified with different samples and instruments by future studies.

Second, agreeableness, maladaptive cognitions, and Internet addiction achieved significant bidirectional associations, which are consistent with the results of previous studies to some degree (Kayiş et al., 2016; Zhou et al., 2017; Hou et al., 2018; Kircaburun and Griffiths, 2018). McCrae and Costa (1997) suggested that agreeable people with characteristics of being highly forgiving and tolerant dislike using force and avoid putting pressure. Previous studies have argued that scores of individuals with Internet addiction are inclined toward using the Internet to avoid negative emotions or relieve stress. Moreover, agreeableness was negatively associated with negative emotions and stress (Suzuki and Matsuda, 2012; Greenidge and Coyne, 2014), which made agreeableness a protective predictor of maladaptive cognitions and Internet addiction. However, spending too much time or money on the Internet increases the levels of social pressure, academic pressure, and economic pressure (while agreeableness prevented individuals from putting pressure); therefore, maladaptive cognitions and Internet addiction negatively predicted agreeableness. These results expanded the cognitive–behavioral model, which indicated only unidirectional associations among personality, maladaptive cognitions, and Internet addiction, whereas bidirectional associations among them were not found. Moreover, the present study’s results suggested that Internet addiction could affect individuals’ agreeableness through maladaptive cognitions, indicating that maladaptive cognitions were also critical factors in the effect of Internet addiction on individuals’ personality.

Third, conscientiousness, maladaptive cognitions, and Internet addiction also achieved significant bidirectional associations, which are in alignment with the findings of previous studies to some extent (Kayiş et al., 2016; Zhou et al., 2017; Hou et al., 2018; Kircaburun and Griffiths, 2018). Conscientiousness people have the characteristics of being cautious, disciplined, planned, decisive, organized, rule and principle abiding, and hardworking (McCrae and Costa, 1997). Therefore, individuals with a higher level of conscientiousness can hardly spend a large amount of time on the Internet because of their traits. However, conscientiousness, maladaptive cognitions, and Internet addiction found execution through vicious circles wherein individuals with higher levels of maladaptive cognitions or Internet addiction were affected by a lower level of conscientiousness, which was an important finding. Moreover, the results suggested that maladaptive cognitions played bidirectional mediating roles in the association between conscientiousness and Internet addiction, indicating that the bidirectional association between conscientiousness and Internet addiction shared the same influential mechanism. These results further expanded the cognitive–behavioral model by demonstrating how maladaptive cognitions are bidirectional mediating factors in the association between personality traits and Internet addiction.

Fourth, neuroticism and Internet addiction also achieved significant bidirectional associations, and no significant associations were found between neuroticism and maladaptive cognitions. Neuroticism has been defined as the loss of emotional balance that is positively associated with negative emotions (such as loneliness and depression; Bunevicius et al., 2008; Buecker et al., 2020). Previous studies have proposed that individuals with negative emotions are more likely to develop Internet addiction than their peers. This is because these individuals tend to use the Internet for relieving negative emotions, obtaining social support, and escaping pressure (Yao and Zhong, 2014; Tian et al., 2017). However, no mediating roles of maladaptive cognitions were found in the association between neuroticism and Internet addiction, indicating that the association between neuroticism and Internet addiction shared different influential mechanisms with other cross-lagged associations in the present study. Different mediating factors may have exerted different effects on the association between the Big Five personality traits and Internet addiction. For example, Zhou et al. (2017) proposed that emotion-focused coping played a greater mediating role in the association between neuroticism and Internet addiction than in the association between other personality traits and Internet addiction. Although the Big Five personality traits were positively related to each other, they exhibited different influential mechanisms on Internet addiction.

Fifth, openness was found to negatively affect individuals’ Internet addiction unidirectionally, which is consistent with the findings of previous studies (Kayiş et al., 2016; Zhou et al., 2017; Hou et al., 2018; Kircaburun and Griffiths, 2018). However, some other studies have identified positive or no association between openness and Internet addiction (Rahmani and Lavasani, 2011; Servidio, 2014). For example, Servidio (2014) suggested that individuals with a higher level of openness demonstrate a higher level of novelty seeking and spend more time on the Internet to seek novel information. By contrast, Kayiş et al. (2016) insisted that both virtual and real activities provide individuals with attractive opportunities to satisfy their curiosity and interest, and that open individuals prefer real-life activities as opposed to virtual ones. Although the results of the current study are consistent with Kayiş’ study, we would like to explain dissimilarities in their results from a new perspective. First, openness was negatively associated with the positive predictors (such as loneliness, depression, and social anxiety) (Bunevicius et al., 2008; Lee et al., 2014; Buecker et al., 2020) of Internet addiction, thereby indicating that openness had a positive effect on the prevention of Internet addiction in individuals. Second, in addition to being novelty seeking or curious, open individuals are independent, which means that these individuals depend less on external things such as the Internet; therefore, openness was a negative rather than a positive predictor of Internet addiction. Similar with agreeableness, the results of the present study also highlighted that Internet addiction could affect openness through maladaptive cognitions, which further contributed to the cognitive–behavioral model. Spending too much time on the Internet reduces an individual’s level of openness, whereas maladaptive cognitions constituted the key driver of this influence.

The present study has several limitations. First, all the studied variables were measured using self-reported questionnaires. Self-reported questionnaires are often associated with measurement errors caused by social desirability and memory recall. Second, the observed variables were used in the study, making it challenging to examine the measurement equivalence of each variable across time. Although the same measurement tools were used to measure the same variables across time, the predictive power of each item and measurement error may have been different across time. Not testing the measurement equivalence of each variable likely introduced more measurement errors in the subsequent data analysis. Third, cross-lagged associations among the Big Five personality traits, maladaptive cognitions, and Internet addiction were examined, which may have introduced the possibility of multicolinearity. Previous studies have examined the association between the Big Five personality traits and Internet addiction by using one model (Zhou et al., 2017; Kircaburun and Griffiths, 2018), whereas the present study examined cross-lagged associations between the studied variables in one model to avoid award excessive complexity to the model. Fourth, because the data were collected after the start of the pandemic, no baseline comparison could be made to support the premise that the present findings constitute unique contributions in this context. Therefore, future studies can compare baseline levels of Big Five personality traits, maladaptive cognitions, and Internet addiction with their corresponding levels during the COVID-19 pandemic. Finally, the present study tested only the bidirectional mediating roles of maladaptive cognitions in the associations between the Big Five personality traits and Internet addiction. Some other studies have found coping style (Zhou et al., 2017), self-liking (Kircaburun and Griffiths, 2018), and family function (Hou et al., 2018) to mediate the association between the Big Five personality traits and Internet addiction; therefore, more mediating factors should be verified through longitudinal studies.

Although this study has several limitations, it makes some critical contributions to relevant research. First, the results of the present study provided important longitudinal evidence to support arguments concerning the association between the Big Five personality traits (especially extraversion and openness) and Internet addiction. The inconsistent conclusions may have been caused by the employment of different samples, different measurement tools, or different data analysis methods. However, both the longitudinal study and meta-analysis provided consistent conclusions that made a difference and suggest that both extraversion and openness were protective factors of Internet addiction. Second, the present study is the first to test the effect of Internet addiction on the Big Five personality traits, suggesting that the association between individuals’ personality traits and Internet addiction was executed in vicious circles wherein Internet addiction was also the predictor of the Big Five personality traits. Third, maladaptive cognitions tended to play bidirectional mediating roles in the association between individuals’ personality traits (such as conscientiousness) and Internet addiction, further expanding the cognitive–behavioral model. In particular, individuals with personality problems developed Internet addiction through maladaptive cognitions, whereas individuals with Internet addiction developed personality problems by developing maladaptive cognitions. Additionally, to test the longitudinal association among the Big Five personality traits, maladaptive cognitions, and Internet addiction during the COVID-19 pandemic was helpful for the family and school’s intervention of Internet addiction during the COVID-19 pandemic. For example, maladaptive cognitions were the key factors in the associations between Big Five personality traits and Internet addiction, which suggested that the family and school could decrease individuals’ Internet addiction with different personality traits by decreasing their maladaptive cognitions about the Internet.

## Data Availability Statement

The raw data supporting the conclusions of this article will be made available by the authors, without undue reservation.

## Ethics Statement

This studies involving human participants were reviewed and approved by the Qingdao University of Science and Technology. The patients/participants provided their written informed consent to participate in this study. The animal study was reviewed and approved by the Qingdao University of Science and Technology. Written informed consent was obtained from the individual(s) for the publication of any potentially identifiable images or data included in this article.

## Author Contributions

YT and YZ wrote the manuscript and data analysis. FL and NQ conducted the data analysis and interpretation of data for the manuscript. PC polished the manuscript and checked the manuscript. All authors contributed to the article and approved the submitted version.

## Conflict of Interest

The authors declare that the research was conducted in the absence of any commercial or financial relationships that could be construed as a potential conflict of interest.
